# Construction of a genetic map and QTL mapping of seed size traits in soybean

**DOI:** 10.3389/fgene.2023.1248315

**Published:** 2023-08-25

**Authors:** Aohua Jiang, Jiaqi Liu, Weiran Gao, Ronghan Ma, Pingting Tan, Fang Liu, Jian Zhang

**Affiliations:** College of Agronomy and Biotechnology, Southwest University, Chongqing, China

**Keywords:** soybean, seed size, genetic linkage mapping, QTL, candidate gene

## Abstract

Soybean seed size and seed shape traits are closely related to plant yield and appearance quality. In this study, 186 individual plants of the F_2_ generation derived from crosses between Changjiang Chun 2 and JiYu 166 were selected as the mapping population to construct a molecular genetic linkage map, and the phenotypic data of hundred-grain weight, seed length, seed width, and seed length-to-width ratio of soybean under three generations of F_2_ single plants and F_2:3_ and F_2:4_ lines were combined to detect the QTL (quantitative trait loci) for the corresponding traits by ICIM mapping. A soybean genetic map containing 455 markers with an average distance of 6.15 cM and a total length of 2799.2 cM was obtained. Forty-nine QTLs related to the hundred-grain weight, seed length, seed width, and seed length-to-width ratio of soybean were obtained under three environmental conditions. A total of 10 QTLs were detected in more than two environments with a phenotypic variation of over 10%. Twelve QTL clusters were identified on chromosomes 1, 2, 5, 6, 8, 13, 18, and 19, with the majority of the overlapping intervals for hundred-grain weight and seed width. These results will lay the theoretical and technical foundation for molecularly assisted breeding in soybean seed weight and seed shape. Eighteen candidate genes that may be involved in the regulation of soybean seed size were screened by gene functional annotation and GO enrichment analysis.

## Introduction

Soybean (*Glycine max* L.) is one of the most economically important crops in the world, which is also used as a model plant for research on legumes. It is a rich source of both edible oil and plant-based protein because of its atmospheric nitrogen fixing capability through a symbiotic interaction with soil microorganisms (Tzen et al., 1993). Soybean is widely grown and consumed globally and constitutes nearly 28% of vegetable oil and 70% of protein meals worldwide (Sulistyo et al., 2021). Soybean is a major oilseed crop that supplies plant protein and oil for humans and animals. Compared with other major crops, such as rice, wheat, and maize, the yield of soybean is approximately 2–3-fold lower. As a result, increasing the soybean yield is an important and urgent task in soybean breeding (Liu et al., 2020a; 2020b). The yield of soybean is a complex trait that is determined by many components, among which seed size is one of the primary indexes. In China, soybean production has continuously declined, with a considerably low yield increase in the past 50 years. Moreover, China imports >80% of soybean for their total domestic use; hence, it is a prerequisite to increase the domestic production of soybean to make the country self-sufficient (Liu et al., 2018). Different yield-related traits are targeted by plant breeders to increase soybean production. In this context, seed weight is one of the most important yield-related traits for increasing seed yield in soybean; however, it is a complex quantitative trait governed by polygenes and is highly influenced by the environment, which makes its selection difficult for plant breeders (Yao et al., 2015).

In crop breeding, seed size is one of the most important agronomic traits that needs to be considered. For instance, the seeds of cultivated crops are usually larger than those of their corresponding wild ancestors, which shows parallel selection (Doebley et al., 2006). Seed size can be described by three main dimensions: length, width, and thickness (Xing and Zhang, 2010). Seed size and shape play a key role in determining seed weight and yield in soybean (Salas et al., 2006; Yan et al., 2017). Seed appearance including seed length (SL), seed width (SW), and seed thickness (ST) as well as seed shape traits such as seed length-to-width (SLW), length-to-thickness (SLT), and width-to-thickness (SWT) ratios affect seed yield (Xu et al., 2011; Liang et al., 2016). Seed size, which is measured with hundred-grain weight (HGW), is a fitness trait that is essential for environmental adaptation (Tao et al., 2017).

Quantitative trait locus (QTL) analysis provides a powerful tool for soybean breeders to search for new sources of variation and investigate the genetic factors underlying quantitatively inherited traits. However, across various genetic backgrounds and conditions, only a few numbers of stable QTL clusters associated with seed and yield-related traits, including seed length (SL), seed width (SW), seed thickness (ST), length-to-width (SLW) ratio, length-to-thickness (SLT) ratio, width-to-thickness (SWT) ratio, and hundred-grain weight (HGW), have been found. Therefore, for effective employment of QTL in marker-assisted breeding, it is essential to find QTLs and confirm them in a variety of backgrounds and conditions. Apuya et al. successfully constructed the first soybean genetic linkage map using F_2_ as the mapping population, which contained 11 RFLP markers, and the experiment demonstrated their distribution across four linkage groups (Apuya et al., 1988). From the soybean database, the results of studies on QTL localization for many traits have been reported. According to the latest database (*SoyBase* (http://www.soybase.org)), 304 QTLs related to HGW have been localized, and 52, 32, and 70 QTLs are related to seed length, seed width, and seed length-to-width ratio, respectively (http://soybase.ncgr.org). In 1996, Mansur detected three QTLs associated with HGW using the RIL population (Mansur et al., 1996). Kulkarni in 2017 identified nine QTLs for HGW, localized on eight linkage groups, using recombinant inbred lines (RILs) constructed from a cross of Williams 82 and PI366121 (Kulkarni et al., 2017). Jun et al. used recombinant self-incompatible line populations derived from a cross between LSZZH and N493 for QTL localization of seed length and seed width and localized eight QTLs related to seed length on six linkage groups and nine QTLs related to seed width on eight linkage groups (Jun et al., 2014). Salas et al. performed QTL localization for seed length using a recombinant self-incompatible population obtained from a cross between Minsoy and Noir l. A total of 13 QTLs associated with seed length were located in six linkage groups (Salas et al., 2006). Kumar et al used vegetable types and seed soy-derived F_2_ and F_2:3_ to map populations. A total of 42 QTLs were identified, distributed on 13 chromosomes (Kumar et al., 2023). Using RILs of 300 individuals populated by the cross derived between soybean PI595843 (PI) and WH as materials, Xu et al. detected a total of 38 QTLs related to HGW, identified four major QTLs, and identified six candidate genes (Xu et al., 2023). Elattaret al used two RIL populations, LM6 and ZM6, to detect 48 mQTLs associated with HGW and 99 mQTLs associated with seed shape traits in 19 soybean chromosomes under four environments (Elattar al., 2021). Chen et al. used an RIL containing 364 individuals of Zhongdou 41×ZYD 02.878 as materials to identify HGW and other traits in soybean. A total of 12 QTLs associated with HGW were identified (Chen et al., 2023).

Until now, there were only a few papers focusing on the mapping of QTL for seed size and shape using the high-density map in various genetic backgrounds of soybean (Karikari et al., 2019). In addition, most of the previous publications did not report the candidate genes for seed shape and seed weight (Zhang et al., 2004; Niu et al., 2013; Kato et al., 2014; Xie et al., 2014; Wu et al., 2018). The present study is aimed at constructing a relatively high-density map and mapping QTL for seed size traits using a population derived from a cross between Changjiang Chun 2 (CJC2) and JiYu 166 (JY166) in three environments, and the results are expected to be useful for marker-assisted selection (MAS) and to improve our understanding of genetic mechanisms underlying seed size traits in soybean.

## Materials and methods

### Plant materials

Changjiang Chun 2 (CJC2) is a high-yielding, high-protein cultivar with larger seeds and a HGW of 23.3 g, which was released in Chongqing, China. JiYu 166 (JY166) is a widely adapted material in China with a HGW of 17.6 g. The significant difference in seed size between the two parents makes the study intuitive and potential. In this study, 186 single plants from the F_2_ population of the cross derived between CJC2 and JY166 were used as the genotyping population.

The F_2_ and F_2:3_ populations were planted in the summer of 2021 and 2022 in Chongqing (21CQ and 22CQ), respectively, and the F_2:4_ population was planted in the winter of 2022 in Yunnan (22YN), China. The F_2_ population was sown with a single plant. F_2:3_ and F_2:4_ families were sown in a single row, with a row length of 1 m, a row width of 0.5 m, and plant spacing of 0.2 m. In addition, all populations were conducted with general field management. The material was harvested after maturity for further examination of seed size traits.

### Trait measurements

Seed shape and hundred-grain weight were evaluated for three generations. The following seed traits were measured using the SC-G software (Wanshen Detection Technology Co., Ltd., Hangzhou, China). Seed length (SL), seed width (SW), hundred-grain weight (HGW), and seed length-to-width ratio (SLW) were determined, and the image analysis method was used for determining the soybean seed traits. Approximately 40 soybean seeds were spread on the white plate of a flatbed scanner (Eloam Technology Co., Ltd., Shenzhen, China). The scanner was set in inverse scanning and positive film mode, 24-bit color and a DPI resolution of 300. The image was processed with the SC-E software (Hangzhou Wanshen Detection Technology Co., Ltd., Hangzhou, China, www.wseen.com). First, the image was converted to a 24-bit grayscale image immediately after scanning and stored in PNG format automatically for further analysis. The image obtained was 3,410 × 2,400 pixels in size. Second, the background was subtracted to remove the effect of background texture, and any overlapped soybean seed was segmented (Zhong et al., 2009). After that, seed parameters were extracted and stored, and the soybean seeds were differently mapped. Finally, the length, width, and HGW of soybean were displayed based on the stored parameters.

The phenotypic data of F_2_ (21CQ), F_2:3_ (22CQ), and F_2:4_ (22YN) generations were statistically analyzed using Excel 2019, SPSS 24.0, and Origin 2019.

### DNA extraction and SSR marker detection

Genomic DNA was extracted from young leaves collected from the F_2_ population of 186 single plants, two parent plants, and F_1_ plants, as described in Zhang et al. (2005). A total of 2,933 SSR primer pairs were synthesized by Biotech Bioengineering Co., Ltd., (Shanghai, China) derived from the soybean database *SoyBase* (http://www.soybase.org/) and Song et al. (2010) (some of these BARCSOYSSR primers were renamed as SWU in this study, as detailed in Supplementary Table S1). PCR amplification was performed as described by Zhang et al. (2002). Primers with polymorphisms between the two mapping parents were used to genotype the single plants of the F_2_ population. The band type identical to CJC2 was recorded as A, the band type identical to JY166 was recorded as B, the heterozygous band type was recorded as H, and the deletion was recorded as U. The results were then gathered for further analysis.

### Map construction and QTL detection

The marker linkage analysis was performed using the mapping software JoinMap 4.0, and the genetic linkage map was constructed with an LOD score of 4.0, a recombination frequency of 0.4, and a converting method of the Kosambi mapping function. The mapping software MapChart 2.2 was used to draw the linkage maps.

The genetic linkage map was used to identify QTLs for seed size traits through multiple QTL mapping methods of MapQTL 6.0. A stringent LOD threshold of the genome-wide association with a *p*-value of 0.05 was used to mark significant QTLs for each trait according to the results from the 1,000 permutation test. Additive effects were defined with respect to the alleles of CJC2. Thus, positive genetic effects indicated the alleles of CJC2 increased the phenotypic trait values, and negative values indicated that the alleles of CJC2 decreased phenotypic values. Those effects with an LOD up to 3.0 were used as an indicator for the presence of QTL.

The QTLs we found were named with the letter q, the trait name, the chromosome number, and the order of QTL identified on the same chromosome. For example, for the QTL denoted as qHGW01.1, q indicates QTL, HGW stands for the trait (hundred-grain weight), 01 shows the chromosome on which the QTL was detected, and 01.1 indicates the order of QTL identified on the chromosome for each trait.

### Candidate gene prediction analysis

The gene and functional annotations in candidate QTLs are obtained on the *SoyBase* (http://www.soybase.org), and the term GO (Gene Ontology) is enriched to analyze the families and sub-families, molecular functions, biological processes, and pathways of the genes in the identified QTLS. Finally, candidate genes for seed size-related functions were screened.

## Result

### Marker polymorphism analysis

Out of 2,933 SSR primer pairs, 518 primer pairs showed polymorphism between the mapping parents. The average polymorphism rate of all primers is 17.7%, and among them, Sat had the highest polymorphism rate of 28.2%, while SWU had the lowest polymorphism rate of 13.0%. A total of 518 pairs of polymorphic primers were used to detect the marker genotypes of the F_2_ population, and 455 marker loci were obtained.

### Genetic map construction

**FIGURE 1:**
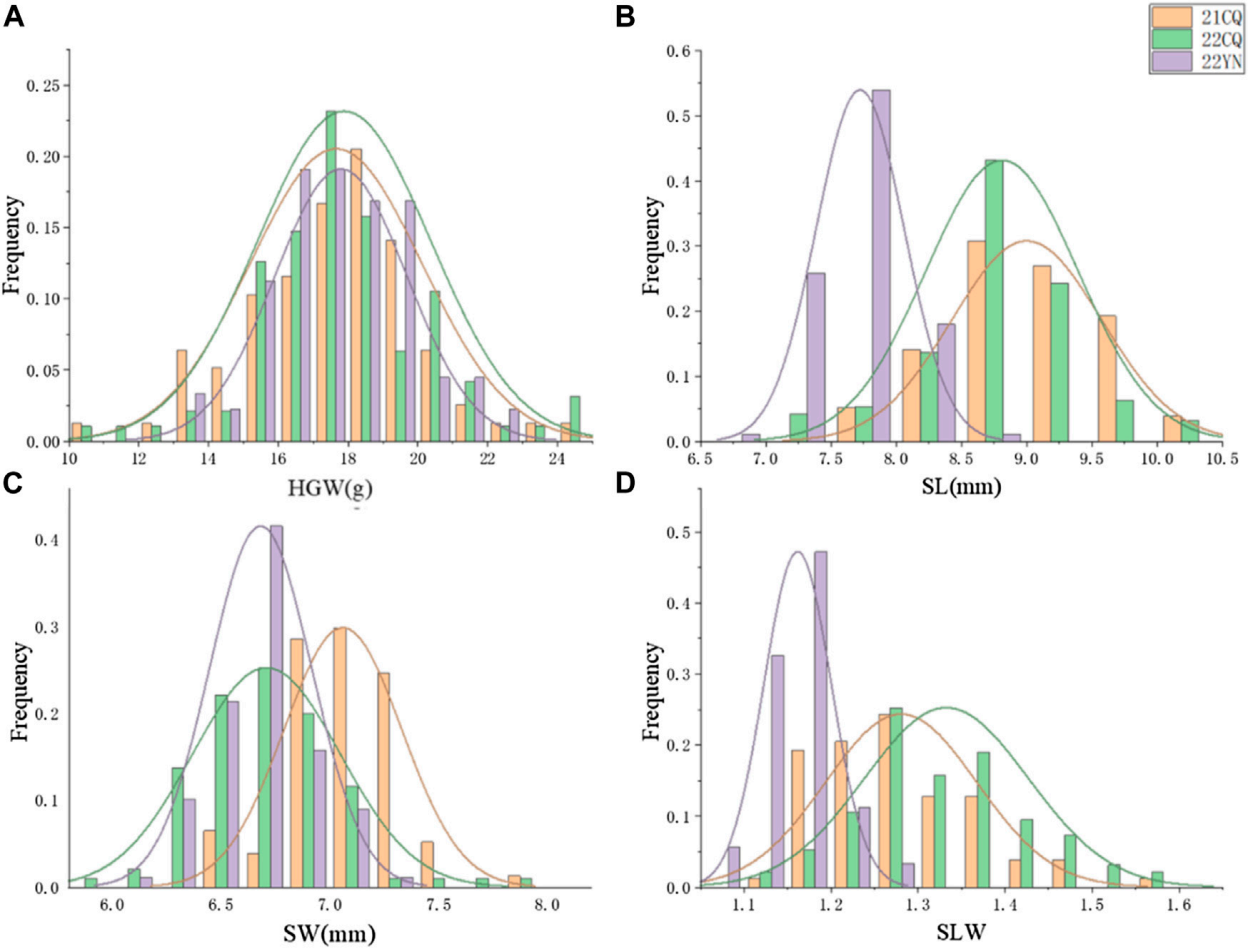
Frequency distributions of seed-related traits in the environment of 21CQ, 22CQ, and 22YN. **(A)** HGW, hundred-grain weight (g); **(B)** SL, seed length (mm); **(C)** SW, seed width (mm); **(D)** SLW, seed length-to-width ratio.

Using the obtained marker loci, a linkage map containing 27 linkage groups and 455 loci was constructed. The map spanned 2799.2 M. The longest linkage group is 217.4 cM, the shortest is 12.2 cM, and the average distance between the markers is 6.15 cM. The maximum number of chromosome 2 markers was 51, and the minimum number of chromosome 16 markers was 10 (Table 1; Figure 1).

**TABLE 1:** Distribution of markers on chromosomes in a map developed from the F_2_ population.

Chromosome	Groups	Markers	Total interval (cM)	Average interval (cM)	Minimum interval (cM)
1	1	32	176.9	5.53	1.0
2	2	51	166.3	3.26	0.3
3	1	26	197.2	7.58	0.6
4	1	18	91.8	5.1	1.1
5	1	22	167.8	7.63	0.5
6	1	23	189.7	8.25	0.1
7	1	27	146.6	5.43	0.6
8	1	15	139.1	9.27	1.7
9	1	17	126.3	7.43	1.1
10	2	19	116.7	6.14	0.6
11	1	15	120.6	8.04	1.7
12	2	13	97.2	7.47	2.2
13	2	23	172.2	7.49	0.2
14	2	24	99.6	4.15	0.5
15	2	16	125.2	7.83	1.3
16	1	10	60.2	6.02	1.2
17	2	20	72.1	3.61	1.5
18	1	35	217.4	6.21	0.6
19	2	32	174.0	5.44	0.5
20	1	17	142.3	8.37	1.6

### Trait phenotype analysis

The results of phenotypic data analysis for the three environments are presented in Table 2. The HGW, seed length, and seed width of CJC2 were higher than those of JY166, and all four traits in the population were segregated to a certain extent, with coefficients of variation ranging from 3.30% to 15.42%. There was transgressive segregation for each trait. The histogram of frequency distribution showed that the four traits were approximately normally distributed in the three environments, which was consistent with the genetic rule of quantitative traits (Figure 1).

**TABLE 2:** Characteristics of seed size traits in the population in three environments.

Trait	Env	Parent		Population							
		CJC2	JY166	Min	Max	Mean	*SD*	Variance	*CV* (%)	Skewness	Kurtosis
HGW (g)	21CQ	24.31	14.87	10.65	22.63	17.49	2.30	5.29	15.42	−0.37	0.18
	22CQ	18.50	16.93	10.47	24.35	17.81	2.39	5.71	13.35	0.03	1.31
	22YN	19.57	18.02	13.60	22.18	17.78	1.87	3.48	10.43	0.14	−0.19
SL (mm)	21CQ	9.71	8.61	7.70	10.29	8.99	0.55	0.31	6.21	0.10	−0.28
	22CQ	9.11	8.38	7.21	10.27	8.81	0.56	0.31	6.30	−0.24	1.37
	22YN	8.13	7.40	6.81	8.44	7.71	0.32	0.10	4.10	−0.07	0.02
SW (mm)	21CQ	7.96	6.47	6.43	7.58	7.05	0.25	0.06	4.51	−0.40	−0.16
	22CQ	6.67	6.61	5.93	7.63	6.69	0.31	0.10	4.65	0.28	0.38
	22YN	6.77	6.69	6.07	7.30	6.68	0.23	0.05	3.44	0.00	0.07
SLW	21CQ	1.22	1.34	1.14	1.55	1.28	0.09	0.01	6.67	0.68	0.15
	22CQ	1.38	1.27	1.15	1.60	1.34	0.09	0.01	6.82	0.47	−0.09
	22YN	1.20	1.11	1.08	1.26	1.16	0.04	0.00	3.30	0.23	−0.10

HGW, hundred-grain weight (g); SL, seed length (mm); SW, seed width (mm); SLW, seed length-to-width ratio; Env, environments. 21CQ, 22CQ and 22YN indicate from 2021 to 2022 in Chongqing and 2022 winter in Yunnan, respectively.

**FIGURE 2:**
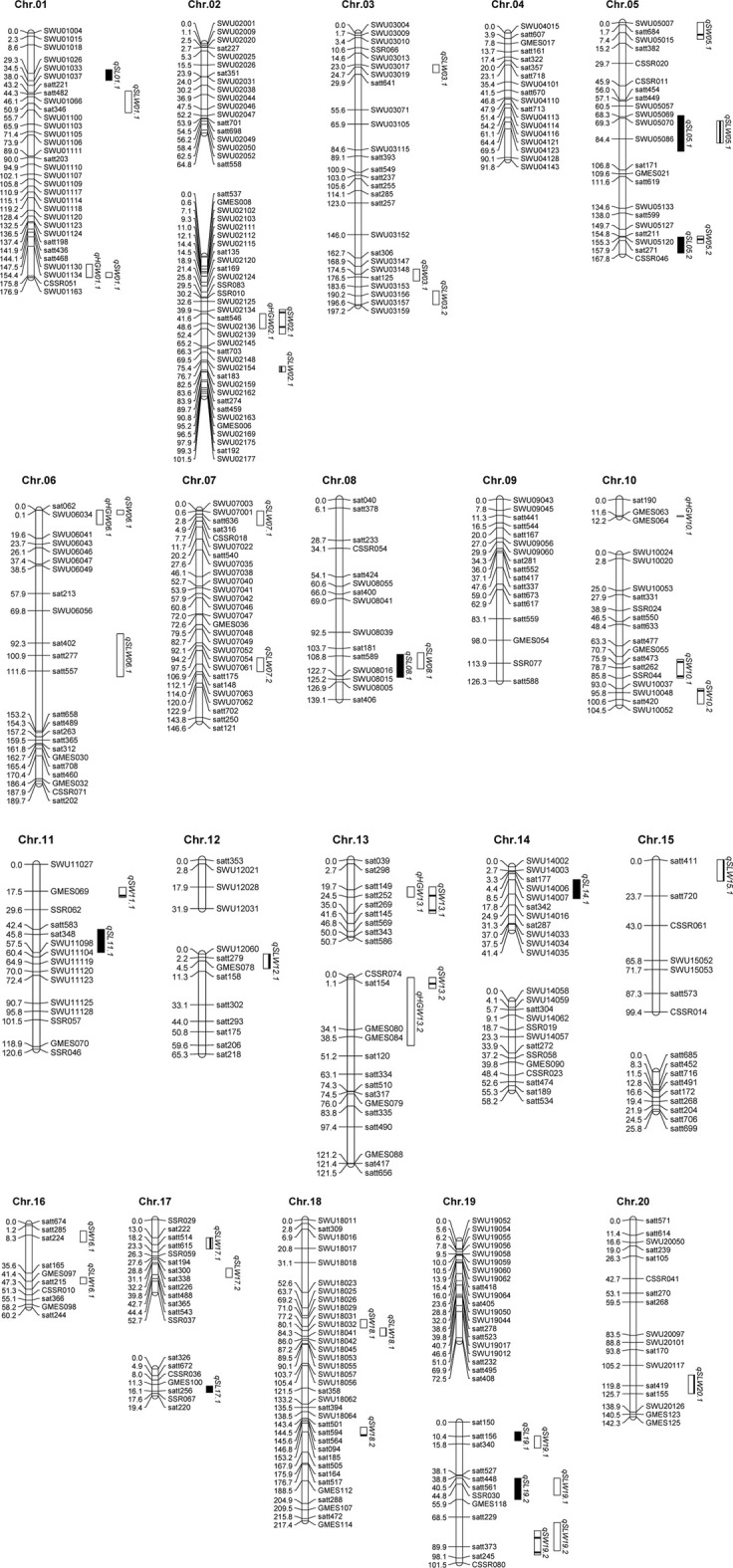
Linkage map and QTL for seed size traits derived from (CJC2 × JY166) population.

Correlation analysis (Figure 2) showed that there was a certain correlation among various traits of seed size. Among the three environments, HGW was positively correlated with SL and SW, while SL was positively correlated with SW. Among them, the correlation coefficient between HGW and SW is the largest and can reach the maximum value of 0.888. Suitable varieties can be selected according to this law in breeding. In addition, since SLW means SL/SW, it is not surprising that SLW is positively related with SL and negatively with SW. HGW is positively related to SL and SW, and it is clear that HGW has a closer correlation with SW than with SL, which indicates a difference between the correlation of HGW and SL and the correlation of HGW and SW. This difference is also different in the three environments. In addition, in the environment of 22YN, the negative correlation between SW and SLW is not that significant, which deserves further detection Table 3.

**TABLE 3:** Correlation analysis of soybean size traits.

Env	Trait	HGW	SL	SW	SLW
21CQ	HGW	1			
	SL	0.672**	1		
	SW	0.762**	0.353**	1	
	SLW	0.175	0.747**	−.0325**	1
22CQ	HGW	1			
	SL	0.580**	1		
	SW	0.797**	0.327**	1	
	SLW	−0.031	0.668**	−0.441**	1
22YN	HGW	1			
	SL	0.521**	1		
	SW	0.888**	0.547**	1	
	SLW	−0.055	0.748**	−0.116	1

^*^ and ** represent significance at the 0.05 and 0.01 probability levels, respectively. HGW, hundred-grain weight (g); SL, seed length (mm); SW, seed width (mm); SH, seed height (mm). 21CQ, 22CQ and 22YN indicate from 2021 to 2022 in Chongqing and 2022 winter in Yunnan, respectively.

### QTLs identified for seed size traits

For hundred-grain weight, six QTLs (Table 4) were identified and mapped on 10 chromosomes, explaining the phenotypic variation from 7.8% to 19.8%. qHGW01.1, qHGW02.1, and qHGW06.1 were identified in two environments, with the maximum phenotypic variation of 16.30%, 14.60%, and 16.40%, respectively. The favorable alleles of six QTL were originated from CJC2.

**TABLE 4:** QTL identified for seed size traits in three environments.

QTL	Env^a^	Chr	Nearest marker	Interval (cM)	LOD	PVE (%)^b^	Additive	Dominance
qHGW01.1	21CQ	01	CSSR051	167.37–176.92	3.00	7.80	1.05	−0.41
	22CQ	01	CSSR051	167.37–176.92	3.60	16.30	1.21	−0.37
qHGW02.1	21CQ	02	SWU02136	41.64–52.41	4.77	12.20	1.29	0.07
	22CQ	02	SWU02136	41.64–52.41	3.18	14.60	1.19	0.07
qHGW06.1	21CQ	06	SWU06034	0.00–10.09	3.45	9.00	1.06	−0.33
	22CQ	06	SWU06034	0.00–10.09	3.61	16.40	1.31	−0.25
qHGW10.1	22CQ	10	GMES064	11.60–12.18	6.11	10.80	0.62	−1.02
qHGW13.1	22CQ	13	satt149	17.73–24.47	4.46	19.80	1.69	−0.39
qHGW13.2	22CQ	13	sat154	0.00–40.46	3.78	17.10	1.39	−0.62
qSL01.1	22YN	01	SWU01026	26.57–34.32	4.54	21.40	0.19	−0.05
qSL05.1	21CQ	05	SWU05070	67.53–93.42	3.16	8.20	−0.24	0.18
qSL05.2	22YN	05	satt211	155.32–166.90	3.46	16.70	−0.74	−1.00
qSL08.1	22YN	08	satt589	107.66–123.74	4.18	19.80	0.17	−0.08
qSL11.1	21CQ	11	sat348	42.37–57.49	3.12	8.10	0.08	0.36
qSL14.1	22YN	14	sat342	8.37–20.78	4.24	20.10	−0.13	−0.17
qSL17.1	22YN	17	satt256	12.26–17.06	4.32	20.40	−0.12	−0.26
qSL19.1	21CQ	19	satt156	7.00–13.45	5.28	13.40	−0.28	0.05
qSL19.2	21CQ	19	SSR030	40.48–55.89	5.31	13.50	−0.26	0.14
qSW01.1	21CQ	01	CSSR051	173.37–176.91	4.45	11.40	0.16	0.08
qSW02.1	22CQ	02	SWU02139	38.64–56.40	4.26	19.00	0.18	0.07
qSW03.1	21CQ	03	SWU03148	170.92–179.46	3.82	9.90	−0.14	−0.09
qSW05.1	21CQ	05	SWU05007	0.00–12.41	3.86	10.00	0.22	−0.04
qSW05.2	21CQ	05	SWU05120	154.69–159.90	6.38	16.00	−0.16	−0.23
qSW06.1	21CQ	06	SWU06034	0.00–3.08	4.74	12.10	0.16	0.07
	22CQ	06	SWU06034	0.00–3.08	3.58	16.30	0.17	−0.02
qSW10.1	21CQ	10	satt262	72.75–85.76	3.61	9.40	0.13	−0.08
	22CQ	10	satt262	76.90–82.71	7.58	31.30	0.23	−0.06
qSW10.2	21CQ	10	SWU10052	96.83–103.56	5.66	14.30	0.20	0.03
	22CQ	10	SWU10048	92.95–100.56	6.65	28.00	0.21	−0.00
qSW11.1	22CQ	11	GMES069	15.00–21.47	3.03	13.90	0.15	0.03
qSW13.1	21CQ	13	satt252	22.72–35.00	4.82	12.30	0.17	−0.09
	22CQ	13	satt149	17.74–29.47	5.44	23.60	0.24	−0.10
qSW13.2	21CQ	13	sat154	0.00–5.13	5.00	12.70	0.11	−0.23
	22CQ	13	sat154	0.00–7.13	3.13	14.40	0.16	−0.12
qSW16.1	21CQ	16	sat224	5.15–13.31	3.83	9.90	0.11	−0.18
qSW18.1	22CQ	18	SWU18031	71.95–78.20	4.00	10.30	0.18	−0.30
qSW18.2	21CQ	18	sat185	149.76–156.16	4.44	11.40	0.01	−0.28
qSW19.1	21CQ	19	sat340	10.00–18.78	3.45	9.00	−0.06	−0.22
qSW19.2	21CQ	19	satt373	86.50–95.91	4.96	12.60	0.18	−0.10
	22CQ	19	satt373	78.50–93.91	4.84	21.30	0.23	−0.01
qSLW01.1	22YN	01	SWU01066	41.95–57.72	4.38	20.70	0.02	0.00
qSLW02.1	22CQ	02	SWU02162	79.66–83.88	4.68	20.70	−0.05	−0.04
qSLW03.1	21CQ	03	SWU03019	23.01–28.69	3.66	9.50	−0.03	−0.06
qSLW03.2	21CQ	03	SWU03156	186.58–196.61	5.16	13.10	0.05	0.00
qSLW05.1	21CQ	05	SWU05070	69.33–81.33	3.60	9.30	−0.04	0.01
	22CQ	05	SWU05086	71.33–85.42	4.70	20.70	−0.06	0.01
	22YN	05	SWU05086	80.33–87.42	3.17	15.40	−0.02	−0.02
qSLW06.1	22YN	06	sat402	85.84–115.63	3.51	17.00	0.02	−0.00
qSLW07.1	22CQ	07	sat316	0.60–10.74	3.69	16.70	0.05	−0.02
qSLW07.2	22YN	07	satt175	102.52–112.09	3.42	16.60	0.02	−0.01
qSLW08.1	22YN	08	satt589	106.66–117.82	3.60	17.40	0.01	−0.02
qSLW12.1	22YN	12	satt279	0.00–9.48	3.02	14.80	0.02	0.01
qSLW15.1	21CQ	15	satt411	0.00–9.00	3.67	9.50	0.04	−0.04
	22CQ	15	satt411	0.00–14.00	3.97	17.80	0.04	−0.04
qSLW16.1	22CQ	16	GMES097	38.65–43.39	3.77	17.00	−0.02	−0.07
qSLW17.1	22CQ	17	satt514	13.02–21.23	4.39	19.50	−0.05	−0.00
qSLW17.2	22CQ	17	satt488	35.17–41.79	4.09	18.30	−0.05	0.02
qSLW18.1	21CQ	18	SWU18032	78.20–83.14	4.01	10.40	−0.03	0.08
	22CQ	18	SWU18032	79.20–84.14	3.43	15.60	−0.02	0.08
qSLW19.1	21CQ	19	SSR030	40.48–52.76	4.84	12.40	−0.04	0.00
	22CQ	19	SSR030	42.48–47.76	4.42	19.70	−0.04	0.03
qSLW19.2	21CQ	19	satt373	72.50–89.92	4.33	11.10	−0.05	−0.01
	22YN	19	satt373	72.50–92.92	3.70	17.80	−0.02	−0.03
qSLW20.1	22YN	20	sat419	112.23–125.70	3.45	16.70	0.02	−0.01

^a^ 21CQ, 22CQ and 22YN indicate from 2021 to 2022 in Chongqing and 2022 winter in Yunnan, respectively.

^b^ PVE, phenotypic variance explained.

For seed length, nine QTLs (Table 4) were identified and mapped on 10 chromosomes, explaining between 8.1% and 21.4% of the phenotypic variation. The favorable alleles of qSL01.1, qSL08.1, and qSL11.1 were derived from CJC2; the favorable alleles of qSL05.1, qSL05.2, qSL14.1, qSL17.1, qSL19.1, and qSL19.2 are derived from JY166.

For seed width, 16 QTLs (Table 4) were identified and mapped on 11 chromosomes, explaining between 9.00% and 31.30% of the phenotypic variation. qSW06.1, qSW10.1, qSW10.2, qSW13.1, qSW13.2, and qSW19.2 were identified in two environments. qSW10.1 and qSW10.2 on chromosome 10 had the largest phenotypic variation of 31.30% and 28.00%, respectively. The favorable genes of qSW03.1 and qSW05.2 were derived from JY166, and the favorable alleles of other QTLs were derived from CJC2.

For the seed length-to-width ratio, 18 QTLs (Table 4) were identified and mapped on 14 chromosomes, explaining between 9.30% and 20.70% of the phenotypic variation. qSLW05.1 was detected in three environments with a maximum phenotypic contribution of 20.70%. qSLW15.1, qSLW18.1, qSLW19.1, and qSLW19.2 were identified in two environments. The additive effects of qSLW06.1, qSLW07.1, qSLW07.2, qSLW08.1, qSLW12.1, qSLW15.1, and qSLW20.1 were positive, while those of the other QTLs were negative.

### Candidate gene prediction within stable QTLs

Through the aforestated QTL localization, this study found that QTLs for multiple traits overlap. A total of 12 QTL clusters were detected on chromosomes 1, 2, 5, 6, 8, 13, 18, and 19, and four QTL clusters with HGW and SW were detected, named qWH. Among those QTL clusters, one QTL cluster named qWH06.1, which was detected stably in two environments (21CQ and 22CQ), was selected for the further detection of candidate genes. The qWH06.1 was located on chromosome 6 from 4953364 bp to 7378025 bp, in which 285 genes were detected. Those genes were then sorted and processed for GO enrichment analysis.

**FIGURE 3:**
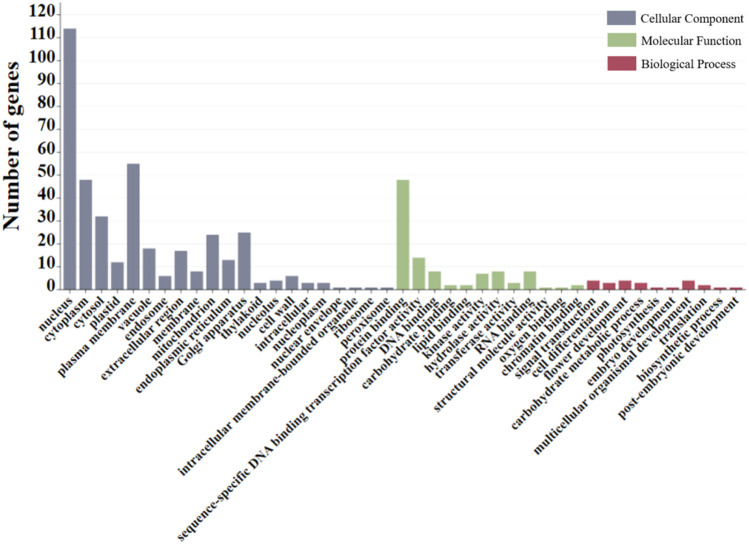
GO term enrichment analysis of the genes located within qWH06.1.

GO enrichment analysis revealed that most genes detected on qWH06.1 are associated with cell composition and molecular function. Most of these genes are concentrated in the nuclear, plasma membrane, protein binding, and other pathways (Figure 3). After gene function annotation screening, a total of 18 candidate genes that may be involved in regulating seed size and weight traits were obtained (Table 5). The 18 genes encode the homeodomain–leucine zipper, the zinc finger transcription factor GATA, the serine/threonine protein kinase, the CXC domain, the cysteine-rich domain, the galactosylgalactosylxyloglucosan 3-beta-glucuronosyltransferase, and others.

**TABLE 5:** Candidate genes identified in seed size QTL regions.

GO ID	Gene	Gene functional annotation
GO:0005730	*Glyma.06g069200*	Helicase-conserved C-terminal domain
GO:0030154	*Glyma.06g086400*	GATA-4/5/6 transcription factors
GO:0030154	*Glyma.06g086600*	Transcription factor HEX, contains HOX and HALZ domains
GO:0005975	*Glyma.06g083800*	Beta-1,3-glucuronyltransferase B3GAT1/SQV-8
GO:0005975	*Glyma.06g091200*	Galactosylgalactosylxylosylprotein 3-beta-glucuronosyltransferase activity
GO:0009790	*Glyma.06g082200*	RNA recognition motif. (a.k.a. RRM, RBD, or RNP domains)
GO:0007275	*Glyma.06g076000*	Proteasome endopeptidase complex
GO:0007275	*Glyma.06g083400*	Tesmin/TSO1-like CXC domain, cysteine-rich domain
GO:0007275	*Glyma.06g092000*	Transcription factor SCREAM2-related
GO:0009058	*Glyma.06g082400*	Cellular amino acid metabolic process
GO:0009791	*Glyma.06g083500*	Protein embryonic flower 1
GO:0030246	*Glyma.06g068100*	Receptor-like serine/threonine protein kinase SD1-8
GO:0008289	*Glyma.06g066600*	Synaptic vesicle protein BAIAP3, involved in vesicle priming/regulation
GO:0016301	*Glyma.06g069900*	Zn-finger in Ran-binding protein and others
GO:0016740	*Glyma.06g067900*	Coactivator CBP, KIX domain-containing protein-related
GO:0003682	*Glyma.06g087700*	Protein SAWADEE homeodomain homolog2
GO:0030154	*Glyma.06g072200*	WD domain, G-beta repeat
GO:0030246	Glyma.06g066400	ER membrane protein complex subunit 7

## Discussion

Soybean seed size traits are mostly quantitative traits controlled by polygenes. At present, there are a lot of QTL mapping studies for quantitative traits in soybean, but most of these QTLs have not been applied to soybean breeding (Csanádi et al., 2001). In this study, the F_2_ generation population derived from the cross between CJC2 and JY166 was used for QTL mapping, and a soybean genetic map with a total length of 2799.2 cM was developed, containing 455 markers with an average map distance of 6.15 cM. There are many genetic markers in this map, which is conducive to subsequent fine mapping and provides conditions for future marker-assisted selection, mining favorable alleles, and exploring related mechanisms of seed development regulation. In this paper, based on the average values of the three environments, the range of HGW between populations was 11.57 g–23.05 g, indicating that there was a large variation in this population, and the coefficient of variation was relatively the largest among the four traits, ranging from 10.43% to 15.42%. A total of six QTLs related to HGW were detected in the population, and all the favorable alleles were from CJC2. Among them, qHGW13.2 has been reported by a previous study (Wu et al., 2020). A total of nine QTLs related to seed length were detected, with phenotypic variation rates ranging from 8.1% to 21.4%. So far, 29 QTLs related to grain length have been published, but few of them have been reported. The QTLs detected in this study were all unreported QTLs. The extremely high temperature in Chongqing in the summer of 2022 affected later seed development, and the addition of generations in Yunnan in the winter of 2022 shortened the growth period of soybean. Both of them had a passive influence on seed width traits. So, it was speculated that seed width growth might occur in late seed development. A total of 16 seed width QTLs were detected, and the phenotypic variation rate ranged from 9.00% to 31.30%. Two of them have been reported. Hina et al. (2020) located a QTL related to seed width on chromosome 1 of soybean, from 49641073 to 51122075 bp, which coincided with qSW01.1 detected in this study, and qSW10.1 detected in this study was also reported in the previous study. The identical results between the QTLs identified in our study and published QTLs for soybean seed size-related traits indicate the accuracy of these QTLs. A total of 46 QTLs have been identified for the first time, and these new QTLs have potential value for the development of improved soybean varieties.

A total of 14 QTLs were detected in more than two environments, and qSLW05.1 was detected in all three environments. Among them, qHGW02.1, qSW06.1, qSW10.2, qSW13.1, qSW13.2, qSW19.2, qSLW05.1, qSLW18.1, qSLW19.1, and qSLW20.1 were detected in more than two environments, with higher than 10% phenotypic variation. These QTLs were identified as stable and dominant QTLs, which could provide a certain reference for further exploration of genes controlling seed size traits (Rahman et al., 2017).

In the present study, we found that there were overlapping QTLs for multiple traits detected, with 12 QTL clusters located on chromosomes 1, 2, 5, 6, 8, 13, 18, and 19, and each QTL cluster was associated with two or more traits related to seed size. QTL clusters may represent gene/QTL linkage or pleiotropic effects from a single QTL in the same genomic region. These QTL clusters can lay a foundation for further mining of target genes controlling seed size. In addition, QTLs for HGW and SW were detected in the same region of multiple chromosomes, and correlation analysis showed that the correlation coefficient between HGW and SW was the largest and strongest. This suggests that there may be a gene with multiple effects coordinating the control of SW and HGW, which could be further explored for the correlation between the developmental mechanisms of SW and HGW.

The QTL intervals related to seed size traits we have detected were compared with that in the soybean public database, and in addition to overlapping intervals with seed size-related traits, many QTLs were found to have overlapping regions with protein content QTL, oil content QTL, days to flowering, and maturity. Therefore, it is hypothesized that genes regulating protein and oil content synthesis or metabolism may be associated with genes regulating soybean seed size. Days to flowering and maturity were suggested by Cober and Morrison (2010) to be directly related to soybean yield, suggesting the potential for common genetic factors for these traits and the need to promote further research on these regions.

Candidate genes are mainly related to cell composition, catalytic activity, transport, metabolism, and cellular processes. *Glyma.06g072200* encodes the WD40 protein that plays an important role in plant growth and development, seed development, and hormone responses, such as the GTS1 (WD40) synergizes with Nop16 and L19e to regulate seed germination and biomass accumulation (Gachomo et al., 2014). Overexpression of GATA encoded by *Glyma.06g086400* not only inhibited growth and biomass accumulation of most phenotypic traits but also altered the expression of some major TFs and pathway genes involved in the secondary cell wall (SCW) and programmed cell death (Ren et al., 2021). *Glyma.06g067900* participates in the encoding of p300 and CBP as transcriptional co-regulators involved in the execution of a wide range of cellular gene expression programs controlling cell differentiation, growth, and homeostasis (Bordoli, 2001). *Glyma.06g069200* encodes the plastid-localized DEAD-box RNA deconjugating enzyme 22, which regulates the accumulation of large quantities of storage products by plants in seeds (Kanai et al., 2013). *Glyma.06g068100* encodes different physiological substrates of threonine/tyrosine protein kinases and their roles in seed oil accumulation (Ramachandiran et al., 2018), which provide energy reserves and nutrients for seed germination and post-germination growth. Forty-three members of the glycosyltransferase family, encoded by *Glyma.06g08380* and *Glyma.06g091200*, were suggested to play an important role in the synthesis of hemicellulosic glucuronic xylan (GX) in plant secondary cell walls (Wu et al., 2010). La is expressed during plant development, and inactivation of *AtLa1* in *Arabidopsis* leads to embryonic lethal phenotypes, in which defective embryos are prevented from developing at early spherical stages (Fleurdépine et al., 2007). Thus, it is hypothesized that La encoded by *Glyma.06g082200* plays a role in seed development. *Glyma.06g083400* is involved in encoding TSO1, which is involved in the maintenance of stable repression of gene expression through cell division and functions to regulate cell proliferation (Reyes and Grossniklaus, 2003), and is thus hypothesized to increase HGW by regulating cellular value addition.

Therefore, based on gene function, GO, and literature search, the 18 aforementioned genes are considered candidates with the potential to regulate soybean seed size traits. However, the exact mechanism remains to be further studied. The markers and candidate genes identified in this study provide important theoretical basis and genetic resources for the improvement of soybean seed size traits.

## Data Availability

The datasets presented in this study can be found in online repositories. The names of the repository/repositories and accession number(s) can be found in the article/Supplementary Material.
